# New insights into the functions and regulation of the transcriptional corepressors SMRT and N-CoR

**DOI:** 10.1186/1747-1028-4-7

**Published:** 2009-04-21

**Authors:** Kristopher J Stanya, Hung-Ying Kao

**Affiliations:** 1 Department of Biochemistry, School of Medicine, Case Western Reserve University and the Research Institute of University Hospitals of Cleveland, 10900 Euclid Avenue, Cleveland, Ohio 44106, USA; 2 Department of Genetics and Complex Diseases, School of Public Health, Harvard University, 665 Huntington Avenue, Boston, Massachusetts 02215, USA

## Abstract

Corepressors are large proteins that facilitate transcriptional repression through recruitment of histone-modifying enzymes. Two major corepressors, SMRT (silencing mediator for retinoid and thyroid hormone receptors) and N-CoR (nuclear receptor corepressor), have been shown to mediate repression associated with nuclear receptors and a myriad of other transcription factors. This review will focus on recent studies on these proteins, including newly discovered physiological roles of the corepressors, their modes of regulation, their roles in antiestrogen-resistant breast cancer and their functions during the cell cycle.

## SMRT and N-CoR are transcriptional corepressors

Control of transcription is mediated by many signaling pathways, including small, non-protein steroids. Steroids control transcription by binding to nuclear receptors (NRs) which in turn modulate transcription of target genes. Studies of nuclear receptor signaling has led to the elucidation of basic mechanisms of transcriptional activation, repression and identification of the specific protein families that control these processes (coactivators and corepressors, [1,2]). In particular, corepressors mediate the active repression of transcription through recruitment of enzymes to post-translationally modify histone tails. Furthermore, corepressors themselves are subject to regulated control of activity, localization and stability through various intercellular pathways. Corepressors are critical for the treatment of certain breast cancers and may also play important roles in the regulation of mitosis.

The first nuclear receptor corepressors identified, SMRT and N-CoR, were isolated in yeast 2-hybrid screens as interacting partners of retinoid X or thyroid hormone receptor (RXR, TR) [3-7]. SMRT and N-CoR share approximately 45% amino acid sequence identity [8] and both are subject to extensive alternative mRNA splicing, generating multiple isoforms [9]. These two corepressors likely share some similar functions while exerting other, distinct influences within cells and organisms. While many interaction partners are shared between the two corepressors, other interaction partners are specific to each corepressor.

### Corepressor domains and stable interacting partners

The corepressors SMRT and N-CoR share similar domain organizations and are believed to be paralogs [8]. Both proteins contain multiple repression domains (RDs), Swi3/Ada2/N-CoR/TFIIID (SANT) motifs [10] and nuclear receptor interaction domains (NRIDs). SANT motifs in corepressors have been shown to be histone binding modules [10,11], although specific mechanisms underlying this are unclear. SMRT contains two NRIDs, while N-CoR contains three NRIDs. The NRIDs in each can be removed by alternative splicing. The RDs likely serve as binding platforms for the various silencing enzymes recruited to repress gene promoters, including the histone deacetylases (HDACs). Thus, both SMRT and N-CoR are part of larger complexes. These corepressor complexes can be considered to be large docking surfaces to tether repression machinery to transcription factors.

Both SMRT and N-CoR have been subjected to extensive biochemical purification to identify core components of their respective complexes. Both complexes contain the same core associated factors, including HDAC3, GPS2 (G protein pathway suppressor 2; X. Cheng and H.Y. Kao, unpublished data) and the transducin β-like factors, TBL1 and TBLR1 [12-16]. These four proteins consistently co-purify together with both SMRT and N-CoR. Interaction of HDAC3 with either the SMRT or the N-CoR complex is thought to promote deacetylase activity on histones [10,13,17]. Other HDACs also interact with SMRT or N-CoR complexes, including class II HDACs 4, 5 and 7 [17-20] and class I HDACs 1 and 2 (through the corepressor mSin3 (mammalian switch independent 3 protein)) [21-23], but their roles in SMRT- and N-CoR-dependent gene repression is unclear. In order to form an active SMRT-HDAC3 complex, association with the TRiC-1 (TCP1 ring complex) chaperone is required [24]. This process is ATP-dependent and TRiC-1 dissociates from SMRT-HDAC3 following complex formation. Although this requirement has only been demonstrated for SMRT complex formation, it is likely that a similar pathway exists for N-CoR complex formation.

### Corepressor-mediated repression

One major function of SMRT and N-CoR is the repression of gene transcription. This function is modulated in part through deacetylation of lysines on histone tails by histone deacetylases contained in large corepressor complexes. Deacetylated histones may serve as preferred binding sites for corepressor complexes in what has been described as a "feed-forward mechanism" [11]. Current models indicate that corepressor complexes initially recognize acetylated chromatin and deacetylates the histone tails. These complexes may then show increased affinity for the deacetylated chromatin, thus enhancing gene repression by increased association. HDAC3 is hypothesized to be the primary histone deacetylase in SMRT/N-CoR complexes. A novel domain termed the deacetylase activating domain (DAD) in both SMRT and N-CoR (located between the two SANT domains) has been shown to promote both the enzymatic activity and binding of HDAC3 [10]. HDAC3 alone targets acetylated lysines on histone H2A and lysines K5 and K12 of H4 *in vitro *[25]; another study reported N-CoR/HDAC3 complexes were specific for H3 *in vitro *[26]. This suggests that corepressor complexes direct the substrate specificity of HDAC3. Extending these studies into mammalian cells, it was shown that SMRT/HDAC3-mediated deacetylation was specific for H4 on the *RARγ2 *gene [27]. Further work may elucidate the specific roles of the various HDACs in corepressor complexes and identify actual target lysine residues on histone tails.

### SMRT and N-CoR have non-overlapping physiological functions

SMRT knockout mice (SMRT -/- mice) have recently been described [28,29]. This gene disruption is an embryonic lethal at approximately embryonic day 16.5 (E16.5) due mainly to defects in cardiogenesis [29]. Specifically, aberrations were observed in ventricular septation and hypoplasia of the ventricular chambers of the heart. The mechanism has been partially delineated as being forkhead box protein 1 (FOXP1) dependent. FOXP1 -/-, SMRT -/- and SMRT +/-/FOXP1 +/- embryos all show similar phenotypes. SMRT and FOXP1 interact in embryonic hearts and colocalize to chromatin to repress target gene transcription [29]. It was further demonstrated that re-expression of α-myosin heavy chain promoter-driven SMRT rescued the SMRT -/- heart defects and the animals survived to birth [28]. However, these rescued animals exhibited significant defects in forebrain development, including major deficiencies in the proportional volumes of dorsal and ventral telencephalon regions. Isolated neural progenitor cells from SMRT -/- animals were prone to differentiate into both neurons and glial cells, indicating that SMRT prevents neuronal stem cell self-renewal. This differentiation effect was shown to be both RAR- and retinoic acid-dependent with the underlying mechanism being repression of the JMJD3 histone demethylase. The absence of SMRT activates JMJD3 which in turn activates genes required for neuronal differentiation by demethylating H3K27 tri-methylation [28].

Similar to SMRT -/- mice, knockout of the N-CoR gene in mice (N-CoR -/- mice) is also an embryonic lethal [30]. However, N-CoR deficient mice typically die by E15.5, one day earlier than SMRT -/- embryos. Observed phenotypes include smaller livers, smaller overall size and anemia due to erythropoietic defects. N-CoR -/- embryos also showed defects in T cell development and lower thymocyte counts. Other defects included major aberrations in nervous system development which was hypothesized to be due to increased neural differentiation. This is similar to SMRT -/- animals, although the underlying mechanisms were not studied in detail. Fibroblasts derived from N-CoR -/- animals displayed altered repression of NR-driven luciferase reporters in transfection assays. Together, these data indicate that N-CoR controls a plethora of important developmental pathways some of which are independent of SMRT.

Recent work from multiple groups has identified several important physiological roles for SMRT and N-CoR using mutational approaches. Two groups, one deleting the NRIDs of N-CoR [31] and the other mutating the NRIDs of SMRT [32], showed that both corepressors are critical for thyroid control of metabolism. Furthermore, SMRT NRID mutant animals were obese due to spontaneous adipogenesis. N-CoR mutant mice that cannot interact with HDAC3 are lean and have abnormal circadian rhythms [33], indicating that corepressor-mediated repression is critical for both metabolism and normal circadian activities. Together, these animal studies indicate that SMRT and N-CoR are critical for normal mammalian physiology.

## Post-translational modifications of corepressors

It is well-established that SMRT and N-CoR repress transcription. Until recently, it was assumed that SMRT and N-CoR had overlapping cellular functions since they share both sequence and functional similarity. However, recent work has demonstrated that these two proteins, while sharing some functions, are regulated in unique ways, including distinct patterns of alternative splicing (reviewed in [9]) and post-translational modifications. This idea is supported by the mouse models mentioned previously, indicating that each corepressor is individually required for distinct pathways critical for normal mammalian development.

### Phosphorylation of corepressors

Several studies investigating the transcriptional regulation of NF-κB-controlled genes have identified a role for SMRT repression and a mechanism that relieves SMRT-mediated repression [34,35]. SMRT has been shown to repress genes such as *ciap-2 *and *IL-8*, which are NF-κB target genes. Initial studies revealed that the IKKα kinase is recruited to chromatin in response to various stimuli where it phosphorylates SMRT at Ser2410. This phosphorylated site serves as a recognition motif for the 14-3-3ε signaling protein, which exports SMRT out of the nucleus leading to proteasome-mediated degradation. This translocation results in activation of the *ciap-2 *and *IL-8 *genes [34].

In addition to IKKα, SMRT is subject to phosphorylation by other kinases, including casein kinase 2 (CK2), epidermal growth factor receptor (EGFR), mitogen-activated protein kinase-related pathways (MAPK) and calmodulin-dependent protein kinase IV (CamKIV). Due to a shift in the electrophoretic mobility of SMRT in response to transforming growth factor β (TGFβ) treatment in several cell lines, various kinase pathways were investigated to identify the source of the shift [36]. CK2, a downstream effector of TGFβ, was found to be the relevant SMRT-targeting kinase. CK2 phosphorylates SMRT on Ser1492 which stabilizes the association between SMRT and NRs and thus enhances repression.

When cells were cotransfected with v-erbB, a constitutively active form of the EGFR (epidermal growth factor receptor), TR-mediated SMRT-dependent repression was abolished with little effect on TR-mediated gene activation [37]. This was due to a loss of interaction between SMRT and TR. It is likely that v-erbB phosphorylates multiple sites on SMRT, as these derepression effects were manifested over the whole protein rather than just the NR boxes that mediate TR binding.

Several studies have identified MAPK signaling as inhibitory to SMRT-mediated repression. The MAPK MEKK1 was shown to phosphorylate SMRT and induce nuclear export of SMRT, thus de-repressing target genes [38]. Further studies established that this signaling was unique to SMRT, as N-CoR remained localized in the nucleus in response to MEKK1 expression [39]. A similar mechanism was shown for SMRT phosphorylation by CamKIV in response to cell stimulation, such as Ca^2+ ^release in neurons [40]. It was further demonstrated that protein phosphatase 1 could dephosphorylate SMRT to induce nuclear retention and promote repression of target genes [40].

SMRT has been shown to co-purify with DNA protein kinase (DNA-PK) and other DNA repair complex components such as Ku70 and Ku80 [41]. Interestingly, Ku70 was shown to recruit SMRT to DNA to repress transcription and this appears to be specific for SMRT and not N-CoR. Furthermore, knockdown of SMRT, but not N-CoR sensitized cells to DNA damage, indicating a potential role for SMRT in DNA damage repair. It is not clear whether DNA-PK phosphorylates SMRT in this pathway.

Few N-CoR-targeting kinases have been identified. Using biochemical methods, DNA-PK was identified in the N-CoR complex and was shown to phosphorylate HDAC3 which inhibits N-CoR-dependent repression [42]. Additionally, Ku70, Ku86 and PARP1 (poly(ADP-ribose) polymerase 1) were also identified in the complex, although not examined in detail. This report, along with the observation that SMRT, but not N-CoR, is required for Ku70-dependent DNA repair [41], suggests that N-CoR either sequesters DNA repair proteins or that complexes containing N-CoR and Ku70 have an as-yet undetermined function. Exploration of this mechanism may reveal further differences between SMRT and N-CoR signaling.

### Degradation of corepressors

**Figure 1:**
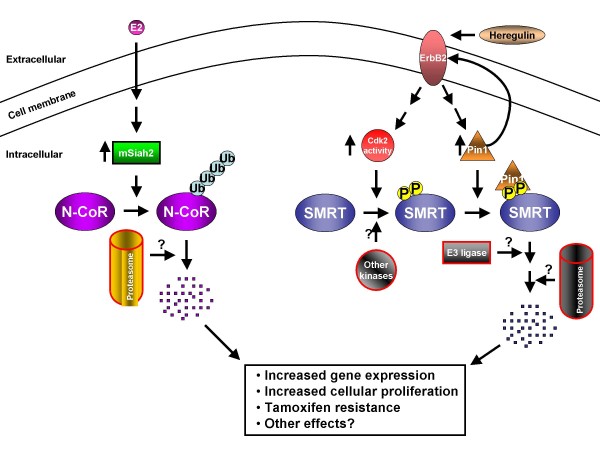
**Degradation of pathways for SMRT and N-CoR**. SMRT and N-CoR are subject to unique degradation pathways. Estrogen (E2) increases mRNA levels of the E3 ubiquitin ligase mSiah2. mSiah2 targets N-CoR for degradation by the proteasome. SMRT is degraded by a distinct pathway. SMRT is phosphorylated by Cyclin-dependent kinase 2 (Cdk2) and potentially other kinases. Phosphorylated SMRT then serves as a substrate for the peptidyl-prolyl isomerase Pin1 which alters the conformation of its substrate. Both Pin1 levels and Cdk activity are increased by the oncoprotein ErbB2; furthermore, Pin1 can increase ErbB2 activity. SMRT is then degraded, likely by subsequent ubiquitination and targeting to the proteasome. Loss of corepressors likely increases transcription of target genes, including some involved in proliferation. Corepressor degradation also likely contributes to tamoxifen resistance.

Several studies have explored the mechanisms controlling the stability of N-CoR and SMRT. Using the N-terminal region of N-CoR as bait in a yeast 2-hybrid screen, the E3 ubiquitin ligase mSiah2 was identified as an N-CoR-interacting protein [43]. mSiah2 was shown to target N-CoR for proteasomal degradation in addition to reversing N-CoR-mediated transcriptional repression (Figure 1). This effect was specific to N-CoR, as no degradation of SMRT was observed. In another study it was observed that estrogen treatment decreased N-CoR protein levels, but not N-CoR mRNA [44]. Estrogen treatment up-regulates both mSiah2 mRNA and protein levels likely resulting in degradation of N-CoR. The degradation could be reversed by treatment with the proteasome inhibitor MG132 or siRNA targeting mSiah2. This mechanism was identified as a novel pathway to activate ER-regulated genes such as 24-hydroxylase.

We have recently identified a novel pathway targeting SMRT for degradation [45]. The peptidyl-prolyl isomerase Pin1 was shown to be a SMRT-interacting protein by yeast 2-hybrid screening. Pin1 binds substrates at phosphorylated serine-proline or threonine-proline dipeptide motifs (pS-P or pT-P) and isomerizes proline motifs from *cis *to *trans *(or *trans *to *cis*). We found that the cyclin-dependent kinase Cdk2 phosphorylates SMRT to generate Pin1 binding sites. Upon binding these sites, Pin1 likely induces conformational changes in SMRT via proline isomerization. Together, Cdk2 and Pin1 promote degradation of SMRT (Figure 1). Neither Cdk2 nor Pin1 coimmunoprecipitated N-CoR, indicating that this pathway is specific for SMRT, as the Siah2 pathway is for N-CoR. Furthermore, both Cdk2 activity and Pin1 levels are positively regulated by the tyrosine kinase receptor Her2/neu/ErbB2. Together, our results indicate that the stability of a corepressor protein can be regulated through a membrane receptor-dependent pathway; this pathway also has important implications in tamoxifen-resistant breast cancers, as discussed below.

## Corepressors and breast cancer

**Figure 2:**
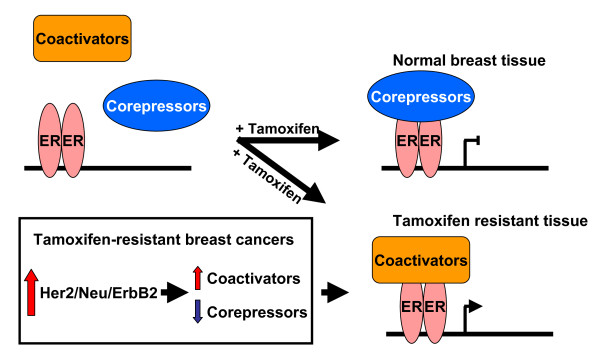
**Corepressors and tamoxifen-resistant breastcancers**. Estrogen receptors (ER) can interact with either coactivators or corepressors. Tamoxifen, a common treatment for ERα-positive breast cancers, induces an ERα conformational change that favors recruitment of corepressors to repress proliferative genes controlled by ERα. However, some breast cancers show ERα recruitment of coactivators even in the presence of tamoxifen, thus resulting in activation of proliferative genes. Many of these tamoxifen-resistant breast cancers overexpress the oncogene ErbB2, which has been shown to increase coactivator levels and decrease corepressor levels, thus altering the delicate ratio between these critical factors.

The majority of breast cancer tumors express ERα. Drugs that target ERα have been a mainstay of breast cancer treatment for nearly 50 years, yet many of the signaling pathways that underlie such treatments remain incompletely understood. This especially pertains to the selective estrogen receptor modulator (SERM) tamoxifen, which remains an important agent in the treatment of ERα-positive breast cancer. Tamoxifen is the major drug used for early stage and advanced premenopausal ERα-positive breast cancer and for prevention of breast cancer in both younger and older women at high risk for developing the disease. Tamoxifen recruits corepressors SMRT and N-CoR to dimerized estrogen receptors [46]. Binding of these corepressors to ERα results in repression of ERα-target genes, including those involved in cell proliferation, through modification of histones and concomitant chromatin remodeling to a more compact state. Thus, corepressor levels are critical for tamoxifen-mediated transcriptional repression and its anti-proliferative activity [47,48]. However, alterations in the balance between coactivators and corepressors can switch tamoxifen from an antagonist to an agonist of ERα-dependent gene expression [49]. Imbalance in the coactivator to corepressor ratio is hypothesized to be a major contributor to tamoxifen resistance in breast cancer (Figure 2). Breast cancer patients that are both ERα-positive and ErbB2-positive are resistant to tamoxifen therapy. Our data strongly suggest that inhibition of Cdk2 and/or Pin1 in ERα-positive and ErbB2-positive breast cancer cells will enhance the anti-tumor activity of tamoxifen.

ErbB2 has been shown to act upstream of both Cdk2 and Pin1. ErbB2 activates the E2F family of transcription factors which in turn increase transcription of both Pin1 [50] and Cyclins A and E [51], both of which are activating factors for Cdk2. Activation of Cdk2 has been implicated in anti-estrogen (tamoxifen) resistance [52]. Pin1 overexpression is prevalent in many different cancers including breast cancer [53] and there is strong correlation between its overexpression and breast cancer in patients [53,54]. Furthermore, Pin1 has recently been shown to stabilize ErbB2 [55], thus completing a positive feedback loop.

ErbB2 is also overexpressed in some ERα-negative breast cancers and prostate cancers. Androgen receptor (AR) is an essential component in androgen-dependent prostate cancer. Similar to ERα, androgen receptor can employ SMRT and N-CoR to repress target genes. Furthermore, overexpression of SMRT or restoring the association of SMRT with AR on AR target genes by MEK inhibitors correlates with the ability of antiandrogens to inhibit prostate cancer cell growth [56]. Therefore, identification of SMRT as a downstream target of ErbB2 that plays a critical role in transcriptional regulation will help develop therapeutic agents for ErbB2-positive cancer patients, such as screening small molecule(s) that increase SMRT protein levels.

## Potential roles for corepressors in cell cycle regulation

While neither SMRT nor N-CoR have been assigned a direct role in regulating the cell cycle, SMRT levels have been reported to fluctuate during mitosis [4]. Knockdown of both SMRT and N-CoR by siRNA increased cell proliferation rates in MCF7 cells [57] while our data indicated SMRT knockdown alone was sufficient to increase proliferation rates in BT474 cells [45]. Furthermore, both Cdk2 and Pin1 are established cell cycle-regulating enzymes [58,59].

Several recent reports have identified a novel role for HDAC3 in cell cycle regulation. In terms of transcriptional regulation, HDAC3 has been shown to repress several critical cell cycle regulators such as the E3 ubiquitin ligase Skp2 [60,61] and several Cdk inhibitors [62]. In addition to transcriptional repression, HDAC3 deacetylates H3 localized at centromeres [63] and facilitates Aurora B recruitment to phosphorylate H3, a required histone modification to proceed through mitosis [64]. HDAC3 has also been shown to localize to the mitotic spindle [65], although its function there remains unknown. Since SMRT is critical for HDAC3 deacetylase activity, we speculate that SMRT may also be important for these activities and thus critical for normal cell cycle progression.

Both whole animal and targeted deletion of HDAC3 suggest a role for this enzyme in cell cycle regulation. HDAC3 -/- MEFs (mouse embryonic fibroblasts) show delayed cell cycling, higher levels of DNA damage and increased apoptosis [66]. Targeted deletion of HDAC3 in either heart [67] or liver [68] resulted in organ hypertrophy that may be attributed to increased proliferation. Together, HDACs and their associated corepressor complexes have been identified as potentially important targets in treating various cancers [69], although further study is necessary to elucidate the mechanisms underlying the roles of these corepressors in cell cycle regulation.

## Concluding remarks

Since the isolation of the transcriptional corepressors SMRT and N-CoR more than ten years ago, the major focus has been their respective roles in transcriptional regulation. These biochemical and molecular studies have established how these two large platform proteins function in many cellular processes. In addition, recent animal and cellular studies have further advanced our understanding of the physiological function and regulation of SMRT and N-CoR. However, we believe that we are only beginning to elucidate the complex regulatory network and biological activity of these two proteins. As SMRT and N-CoR play important roles in animal development, homeostasis and are also associated with human diseases, elucidation of the mechanisms and upstream signaling controlling their activities is critical.

Several important avenues remain unexplored. For example, if HDAC3 is a stable component of SMRT and N-CoR corepressor complexes and a critical regulator of the cell cycle, are SMRT or N-CoR also critical for HDAC3-mediated cell cycle progression? Is SMRT or N-CoR regulated in a cell cycle-dependent manner? How do SMRT and N-CoR contribute to DNA damage repair and how do post-translational modifications control stability of these two corepressors? These questions warrant future investigation.

## Competing interests

The authors declare that they have no competing interests.

## Authors' contributions

KJS and HYK drafted the manuscript.
